# Case of Ulceration Following Venæsection

**Published:** 1806-04

**Authors:** 


### Case of Ulceration following Venæsection.

John Edwards, ætat. 18, of a plethoric habit, was bled in the basilic vein—the lancet gave little pain, the blood flowed in a full stream, and when stopped, the orifice was immediately closed, and bound over in the usual manner. About three days after this, the orifice inflamed, became painful, and then ulcerated; the inflammation soon became diffused, while the ulcerated part healed. In a short time the inflammation occupied a considerable surface, terminating near the axilla above, and at the extremities of the fingers below; at the same time the limb swelled was very hot, tense, and painful. During the progress of these symptoms, all the phenomena of fever ensued; in consequence of which, an antiphlogistic regimen, and a solution of acetite of lead (to the limb) were ordered. However, the patient at this time, refusing all internal remedies, the whole was left to local ones, which seemed to relieve the part; but the state of the system continued much the same.

About ten days from the commencement of fever, the constitution was so debilitated, that tonic medicines were strongly indicated, and with this view, bark, opium and vinous drinks were prescribed. The whole limb soon became œdematous, and afterwards two dense cords appeared; one above, the other below the first seat of the injury; these followed the direction of the vein, united in the middle, and at each end formed a tumour, which upon being pressed felt painful, but by no means acutely so. To these, warm cataplasms were applied and frequently renewed.

By persisting in this plan, joined with the power of the tonic remedies, the constitution became vigorous, the local symptoms disappeared, and the muscles gained their powers.

As this case is rather curious and uncommon, it may be proper to offer a few remarks upon it.—The patient never was bled before, and therefore the cause may be difficult to investigate. However, three causes may be assigned, the most probable of which, let us endeavour to ascertain. The first is the puncture of a tendon; the second, the exposure of an internal cavity; and the third, the puncture of a nerve.

That it did not arise from the first of these is probable, because the vein was superficial, and no tendinous structure ture contiguous; besides, had the lancet passed too deep, the artery must have suffered, as it passed beneath the vein, and seemed to pulsate very strongly. That it did not arise from simple exposure is also probable, for during the time the blood flowed, no air could possibly have access, and when it ceased to flow, its ready admission was prevented by the orifice being immediately closed and bound over; and I should think, if it could be attributed to this cause, it would not be so very rare an occurrence, especially when we consider the very little care that is generally taken, in applying the lips of the wound together. The most probable cause, is the puncture of a sub-cutaneous nerve. Every one will allow the probability of this, when they consider the dreadful symptoms that have been produced by the puncture of a small nerve, in the extremity of the finger. If the smallness of the nerve should be objected to, let us reflect on the violent symptoms sometimes present during dentition. These have been prevented by the transverse division of the nerve. Might not this be practised with the same success in inflammation of the arm after bleeding, when it originates from the puncture of a nerve?

Having now taken notice of the three most probable causes, and having mentioned that the local symptoms took their rise from a local cause, let us consider some further circumstances, with a view to illustrate another opinion; that is, of their being constitutionally local; by this, I mean that there may be a predisposition.—In behalf of this, I will mention, that the father was a stout plethoric man, and was bled some years ago: his arm inflamed, and he says, underwent the same disease as that of his son's. This would seem to point further, and to show an hereditary taint; but how far this existed, is not to be easily ascertained. Another argument, seemingly strong, might be deduced from the following fact. A young woman, apparently healthy, was bled in the basilic vein; this was followed by great inflammation. She said, that, every time she was bled, the same symptoms followed; these remarks would seem to favour a predisposition, but as this is very improbable, I will offer a few remarks in contradistinction to it.

First I will observe, that inflamed arms after bleeding, as often occur in constitutions apparently healthy, as in those that seem to be diseased; and in addition to this, I will add, that one arm of a healthy person shall lose blood and these symptoms arise, while blood drawn from

 the the other arm, of the same person, shall not produce a single symptom. Of this I have seen a striking instance; but I remarked, that the person complained of pain, during venæsection, in the arm that afterwards inflamed, but not in the least in the one which remained sound. From these it would appear, that some subcutaneous nerve was punctured, and the constitution not in fault; the primary symptoms being quite local, while all those that were universal, seemed only to be the consequence of sympathy.

